# CXCR4-targeted Theranostics in Hematooncology: Opportunities and Challenges

**DOI:** 10.1055/a-2194-9965

**Published:** 2024-01-08

**Authors:** Rudolf Werner, Alexander Haug, Christian Buske, Simon Heidegger, Anna L. Illert, Florian Bassermann, Peter Herhaus, Andreas Buck, Johannes Duell, Max S Topp, Sabrina Kraus, Hermann Einsele, Constantin Lapa, Markus Raderer, Georg Lenz, Stefan Habringer, Bastian von Tresckow, Ulrich Keller

**Affiliations:** 1 Department of Nuclear Medicine, University Hospital Würzburg, Würzburg, Germany; 2 Clinical Department of Nuclear Medicine, University Hospital AKH Vienna, Wien, Austria; 3 27197 Institute of Experimental Cancer Research, Ulm University Hospital, Ulm, Germany; 4 9184 Department of Medicine III, School of Medicine, Technical University of Munich, Munchen, Germany; 5 Department of Hematology and Oncology, Freiburg University Hospital, Freiburg, Germany; 6 Internal Medicine II, University Hospital Würzburg, Würzburg, Germany; 7 26522 Nuclear Medicine, Faculty of Medicine, University of Augsburg, Augsburg, Germany; 8 Department of Internal Medicine I, Medical University of Vienna, Vienna, Austria; 9 39069 Department of Medicine A - Hematology, Oncology and Pneumology, University Hospital Münster, Münster, Germany; 10 14903 Department of Hematology, Oncology and Cancer Immunology, corporate member of Freie Universität Berlin and Humboldt-Universität zu Berlin, Charite Universitatsmedizin Berlin, Berlin, Germany; 11 39081 Department of Hematology and Stem Cell Transplantation, West German Cancer Center, University Hospital Essen, Essen, Germany

**Keywords:** CXCR4, chemokine receptor, theranostics, marginal zone lymphoma, T-cell lymphoma, [68Ga]Ga-PentixaFor

## Abstract

C-X-C motif chemokine receptor 4 (CXCR4) is overexpressed in a multitude of cancers, including neoplasms of hematopoietic origin. This feature can be leveraged by a theranostic approach, which provides a read-out of the actual CXCR4 expression in vivo, followed by CXCR4-targeted radioligand therapy (RLT) exerting anti-cancer as well as myeloablative efficacy. In a recent meeting of hematooncology and nuclear medicine specialists, statements on the current clinical practice and future perspectives of this innovative concept were proposed and summarized in this opinion article. Experts concluded that i) CXCR4-directed [68Ga]Ga-PentixaFor PET/CT has the potential to improve imaging for patients with marginal zone lymphoma; ii) CXCR4-targeted RLT exerts anti-lymphoma efficacy and myeloablative effects in patients with advanced, treatment-refractory T-cell lymphomas; iii) prospective trials with CXCR4-based imaging and theranostics are warranted.

## Introduction

Theranostics combines molecular imaging and therapy through applying identical or related radiolabelled molecules to specifically detect and damage cancerous tissues 1 . While such radiopharmaceuticals are now routinely applied for solid tumours 2 3 , recent years have witnessed an increasing use for hematopoietic malignancies, in particular by targeting C-X-C motif chemokine receptor 4 (CXCR4) 4 . In the course of the annual meeting of the German Society for Haematology and Medical Oncology held in October 2022, experts met to discuss recent developments in the field of CXCR4-based theranostics and to devise some basic statements on potential clinical applications and future perspectives of this concept.

In this manuscript, we present these statements in detail, along with a brief overview on CXCR4-directed molecular imaging and therapy.

### Expert panel statement I: CXCR4-directed molecular imaging may be useful for marginal zone lymphoma patients

#### CXCR4-directed Molecular Imaging

**Fig. 1:**
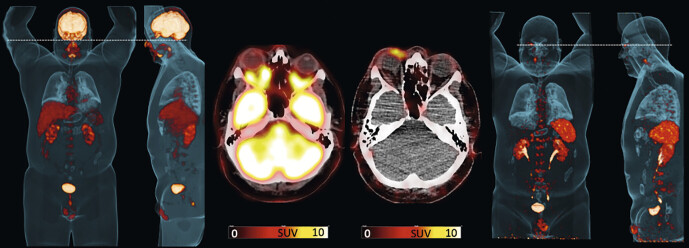
Maximum intensity projections of PET/CTs using [18F]FDG (leftmost panels) and [68Ga]Ga-PentixaFor (rightmost panels) in a subject with orbital marginal zone lymphoma. The middle panels show PET/CT images of axial sections of lymphoma manifestation using [18F]FDG (bottom) and [68Ga]Ga-PentixaFor (top). On [18F]FDG, MZL manifestation is not visible due to the physiological high uptake of the PET probe in brain tissue, whereas the [68Ga]Ga-PentixaFor-based images enables detection of the manifestation in the periorbital area. Data from same patient but different images as in Buck et al, J Nucl Med 2023 Jul;64(7):1009–1016 4 .

CXCR4 upregulation in varying tumour entities render it as an attractive target to identify cancerous tissue 5 , which can be exploited using CXCR4-directed radiotracers, including the cyclic ^68^ Ga-labelled CXCR4-binding pentapeptide CPCR4.2 ([ ^68^ Ga]Ga-PentixaFor) 6 . Relative to the most widely used PET radiotracer 2-deoxy-2-[ ^18^ F]fuoro-D-glucose ([ ^18^ F]FDG), [ ^68^ Ga]Ga-PentixaFor often achieved equal, sometimes superior or at least complementary diagnostic performance in nuclear oncology 7 8 9 10 . Accordingly, [ ^68^ Ga]Ga-PentixaFor has been extensively tested to identify sites of disease in patients with solid tumours and haematological malignancies, providing promising results for marginal zone (MZL, Fig. 1 ) and T-cell lymphoma (TCL) 9 11 . Beyond an improved read-out relative to the reference radiotracer [ ^18^ F]FDG, however, [ ^68^ Ga]Ga-PentixaFor also allows for quantification of CXCR4 expression in lymphoma manifestations in vivo, thereby identifying patients that could therefore be eligible for CXCR4-directed radioligand therapy (RLT) using the theranostic, β-emitting twin [ ^177^ Lu]Lu- or [ ^90^ Y]Y-PentixaTher 12 .

#### CXCR4-directed PET/CT for Marginal Zone Lymphoma

The most comprehensive approach investigating the use of [ ^68^ Ga]Ga-PentixaFor for imaging of solid and haematological neoplasms was conducted in a multi-centric retrospective analysis from Vienna and Würzburg Universities 10 . [ ^68^ Ga]Ga-PentixaFor PET/CTs were conducted in 690 patients and almost 70% showed radiotracer accumulation in sites of disease which were substantially higher than physiological background activity. In this regard, the highest uptake was observed for multiple myeloma (MM), mantle cell lymphoma (MCL) and MZL 10 . Duell et al. then imaged 22 newly diagnosed MZL patients with chemokine receptor PET/CT and compared those findings with conventional, guideline-compatible diagnostic work-up (including [ ^18^ F]FDG PET/CT, bone marrow biopsy, and endoscopy) 9 13 . In 16 of 18 PET-guided biopsy samples, imaging results could be confirmed upon CXCR4 immunohistochemistry. [ ^68^ Ga]Ga-PentixaFor PET/CT had a significant impact on staging according to modified Ann Arbor classification in nearly 50% (mostly upstaging) and, importantly, on treatment decision in one third of the patients 9 . A more comprehensive investigation involving 100 MZL patients recently reported comparable results 14 . [ ^68^ Ga]Ga-PentixaFor uptake was observed in 78% of the patients, and again, in almost half of the subjects, this led to an upstaging using Ann Arbor classification. In addition, an elevated CXCR4-PET signal showed a trend towards shorter progression-free survival, suggesting that [ ^68^ Ga]Ga-PentixaFor may also represent a prognostic biomarker with regard to disease progression and need for improved/intensified treatment 14 .

As a sub-form of MZL, mucosa-associated lymphoid tissue (MALT) lymphomas are diagnostically challenging as they often lack specific symptoms and are associated with multi-organ involvement 15 , but also known to overexpress CXCR4 16 . Haug and colleagues examined MALT lymphoma patients with a wide range of organs affected using [ ^68^ Ga]Ga-PentixaFor PET/magnetic resonance imaging (MRI) and found an intense uptake in 33/36 patients, while three negative cases having undergone surgery for their orbital MALT lymphomas prior to imaging 17 . Another prospective study used [ ^68^ Ga]Ga-PentixaFor PET/MRI to evaluate treatment responses after first-line *Helicobacter pylori* eradication in 26 gastric MALT lymphoma patients and compared CXCR4-based imaging findings with a control group of 20 subjects without lymphoma 18 . [ ^68^ Ga]Ga-PentixaFor PET/MRI yielded a 100% detection rate, as confirmed in time-matched gastric biopsies. Furthermore, neither false-positive nor false-negative results were obtained, and overall, between 93% and 100% accuracy, sensitivity, specificity, positive and negative predictive values for the detection of residual gastric disease were determined.

At present, diagnostics of MZL and MALT lymphoma are rather complex due to the heterogeneity of the disease and often cumbersome for the patients when multiple biopsies and possibly endoscopies are required 13 . This, together with the provided benefit of CXCR4-targeted PET/CT as summarized above, led the experts to suggest that [ ^68^ Ga]PentixaFor PET might serve as a valuable diagnostic tool for MZL including MALT lymphoma, and should be further developed in prospective clinical trials.

### Expert panel statement II: CXCR4-based theranostics may be useful in advanced, treatment-refractory T-cell lymphoma

#### CXCR4-directed Radioligand Therapy

After having identified CXCR4 expression using [ ^68^ Ga]Ga-PentixaFor PET, the positron emitter can be exchanged with an α- or β-emitting radionuclide that exerts direct cell damaging effects, e.g., [ ^177^ Lu]Lu-/[ ^90^ Y]Y-PentixaTher 19 . Of note, this theranostic counterpart exhibits not only anti-lymphoma, but also myeloablative effects due to targeting the hematopoietic stem/progenitor/cell (HSPC) compartment, which may be desired for selected patients affected with hematopoietic neoplasms to prepare for hematopoietic stem cell transplantation (HSCT) 12 . As a biological rationale, expression of CXCR4 is not only pronounced in lymphoma sites, but also in cells of the bone marrow 20 . Thus, CXCR4-mediated RLT then also allows targeting of the HSPC niche beyond targeting manifestations of the underlying CXCR4-positive disease 12 .

#### CXCR4-directed treatment for T-Cell Lymphoma

**Fig. 2:**
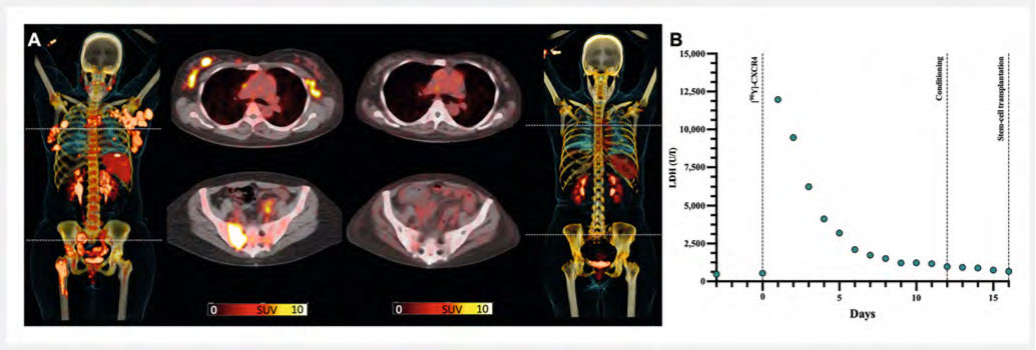
Complete metabolic response after CXCR4-directed radioligand therapy (RLT) in a patient affected with advanced, treatment-refractory T-cell lymphoma. After RLT, conditioning therapy and allogeneic haematopoietic stem cell transplantation were conducted, leading to successful engraftment. ( **A** ) Maximum intensity projections of CXCR4-targeted [68Ga]PentixaFor PET/CTs before (leftmost panel) and 4 months after (rightmost panel) RLT with [90Y]PentixaTher. Prior to therapy, multiple CXCR4-positive foci on transaxial PET/CTs were visible, including lymphonodal, peritoneal and bone manifestations (middle panels, left), whereas imaging four months after RLT showed a complete response (middle panels, right). ( **B** ) Time course of lactate dehydrogenase (LDH, in U/l) serving as a surrogate marker of induced lymphoma damage. An initial peak of LDH was followed by a rapid decrease, indicating an immediate response to CXCR4-directed RLT independent of conditioning therapy. Data from same patient but different images as in Buck et al, J Nucl Med, 2023 Jan;64(1):34–39 11 .

In peripheral TCL, a wide range of first-line treatment regimens exists, which are frequently not able to provide long-term disease control or cure. Targeted and more effective therapeutic regimens are available for patients with CD30 expressing TCLs 21 . However, CD30 is expressed only in approximately half of the patients 22 . In selected patients, which are refractory to standard therapeutic regimen (chemotherapy ± CD30 antibody ± high-dose therapy and autologous HSCT), allogeneic HSCT involving cytotoxic bone marrow ablation (myeloablation) may be indicated 23 . In this regard, CXCR4-directed RLT using [ ^90^ Y]Y-PentixaTher may also provide relevant myeloablative efficacy 11 . For instance, in four TCL patients with advanced disease having exhausted all previous treatment options, a retrospective examination with CXCR4-PET/CT was followed by CXCR4-targeted RLT combined with conditioning regimen and HSCT. One of these patients developed tumour lysis syndrome and transient grade 3 kidney failure, while one patient died more than two weeks after RLT after developing septicaemia. Partial metabolic response was observed in one and complete metabolic response in the two other patients (with one subject also treated with additional radioimmunotherapy; Fig. 2 ) 11 . As such, CXCR4-mediated RLT may serve as an effective conditioning therapy for HSCT with concurrent anti-lymphoma activity in treatment-refractory TCL in advanced disease setting.

### Expert panel statement III: Prospective trials are required to provide additional data and guidelines on CXCR4-directed imaging and therapy in malignancies of the hematopoietic origin

Considering the wide range of highly promising but often preliminary or only retrospectively assessed data, the experts concluded that there is a need for prospective studies investigating the use of CXCR4-directed radiopharmaceuticals for both imaging and therapy. At present, prospective trials for CXCR4-directed RLT are in preparation, aimed at investigating the activity, tolerable dose and side effects of [ ^90^ Y]Y-PentixaTher in patients with recurrent or refractory primary or isolated secondary central nervous system lymphoma, (PTT101, EudraCT No. 2021–002364–43), multiple myeloma (MM) and other lymphoma subtpes (COLPRIT trial, EudraCT No. 2022–002989–33) 24 25 . Another registered study investigates [ ^68^ Ga]Ga-PentixaFor PET/CT for initial staging and therapeutic monitoring for MM (NCT04561492) 26 . In addition, a basket trial enrolling approximately 20 patients will also broaden the diagnostic experience on [ ^68^ Ga]Ga-PentixaFor in patients with varying hematopoietic (pre)neoplasms, including monoclonal gammopathy of undetermined significance, smoldering MM and Non-Hodgkin lymphoma (NCT05093335) 27 . Moreover, a phase III study in MZL patients will compare [ ^68^ Ga]Ga-PentixaFor with [ ^18^ F]FDG PET/CT in 148 patients examined in 28 participating European centres 28 . Table 1 provides an overview of planned and currently recruiting studies on CXCR4-directed imaging and therapy in patients affected with malignancies of the hematopoietic system.

**Table:** **Table 1** Overview of selected prospective clinical trials on CXCR4-directed Theranostics focusing on Haematological Malignancies.

Purpose	Study Abbreviation*	Registration No.	Brief Description	Status
Diagnosis	PentiMyelo	NCT04561492	Phase I/II study using [ ^68^ Ga]Ga-PentixaFor PET/CT for staging and therapeutic monitoring of multiple myeloma (MM) patients scheduled for first line treatment 26	Recruiting
		NCT05093335	Phase I/II study using [ ^68^ Ga]Ga-PentixaFor PET/CT in treatment-naïve, therapy-refractory or relapsed patients with proven monoclonal gammopathy of undetermined significance, smoldering MM, or Non-Hodgkin lymphoma 27	Recruiting
	PTF301 (LYMFOR)	EU CT No 2022–500918–25	Phase III study using [ ^68^ Ga]Ga-PentixaFor PET/CT relative to [ ^18^ F]FDG PET/CT for staging of patients with marginal zone lymphoma 28	Planned
	PENTI-MIDAS	NCT05321862	Phase I/II study using [ ^68^ Ga]Ga-PentixaFor for staging and assessment of minimal residual disease in MM patients eligible for hematopoietic stem cell transplantation [29]	Not yet Recruiting
Therapy	COLPRIT	EudraCT No. 2022–002989–33	Phase I/II study evaluating CXCR4-directed radioligand therapy (RLT) in advanced lymphoproliferative disease 24	Planned
	PENTILULA		Phase I/II study evaluating RLT with [ ^177^ Lu]Lu-PentixaTher in patients with relapsed or refractory CXCR4+ acute leukemia	Planned
	PENTALLO		Phase I study testing RLT with [ ^177^ Lu]Lu-PentixaTher prior to allograft transplantation in acute myeloid leukemia/acute lymphocytic leukemia patients	Planned
	PTT101	EudraCT No. 2021–002364–43	Phase I/II dose escalation study to evaluate safety, tolerability, biodistribution and efficacy of [ ^90^ Y]Y-PentixaTher for recurrent or refractory primary or isolated secondary central nervous system lymphoma 25	Planned
*if available.				

As agreed by the expert panel, these studies and others yet to be conceived should ultimately allow for assessing the most appropriate applications of the theranostic concept.

## Conclusions

CXCR4-directed theranostics using the PET agent [ ^68^ Ga]Ga-PentixaFor and its therapeutic twin [ ^177^ Lu]Lu-/[ ^90^ Y]Y-PentixaTher has been extensively investigated in recent years. Based on these results, its diagnostic use appears to be promising in patients with MZL including MALT lymphoma. For therapeutic applications, anti-lymphoma efficacy exerted by CXCR4 RLT may be useful in advanced and treatment-refractory TCL. Nonetheless, data from prospective studies are required to allow for incorporation of this theranostic strategy into clinical practice.
